# Audit on Current Practice of Rapid Sequence Induction and Intubation of Anesthesia in the University of Gondar Hospital, Northwest Ethiopia, 2018

**DOI:** 10.1155/2019/6842092

**Published:** 2019-09-22

**Authors:** Mamaru Mollalign, Amare Hailekiros Gebreegzi, Habtamu Getinet, Seid Adem

**Affiliations:** University of Gondar, Gondar, Ethiopia

## Abstract

**Background:**

In patients who are liable to the risk of pulmonary aspiration, airway control is the primary and first concern for the anesthetists both in emergency and elective surgical procedures. Rapid sequence induction is universally required in any occasion of emergent endotracheal intubation needed for unfasted patients or patients' fasting status is unknown.

**Methods:**

institutional-based prospective observational study was conducted from December 2017 to January 2018 in all elective and emergency adult or pediatric patients with a risk of pulmonary aspiration who were operated under general anesthesia with rapid sequence induction and intubation during the audit period.

**Result:**

A total of 35 patients were operated during the study period. Of these, 31 (88.57%) patients were adults and 4 (11.43%) patients were pediatrics. Most of the patients were emergency (29 (82.857%)), and the rest were elective (6 (17.142%)).

**Conclusion:**

Most anesthetists were good at preparing all available monitoring and drugs, making sure that IV line is well-functioning, preparing suction with a suction machine, preoxygenation, application of cricoid pressure, and checking the position of the ETT after intubation was performed. Preparing difficult airway equipment during planning of rapid sequence induction and intubation, giving roles and told to proceed their assigned role for the team, attempt to ventilate with a small tidal volume, and routine use of bougie or stylet to increase the chance of success of intubation needed improvement.

## 1. Introduction

In patients who are liable to the risk of pulmonary aspiration, airway control is the primary and first concern for the anesthetists both in emergency and elective surgical procedures and in any occasion of need of airway protection [1].

Rapid sequence intubation and induction of anesthesia is a very fundamental skill of anesthesia practice during encountering patients who are at risk of pulmonary aspiration. It is commonly used to prevent regurgitation and vomiting of gastric contents at aiming of protecting the airway. To optimize patient outcome and to reduce risk of hypoxia, currently the modified technique of rapid sequence induction/intubation is practiced in certain clinical circumstances [2, 3].

The modified technique of rapid sequence induction/intubation is the use of pharmacological prophylaxis, preoxygenation, application of cricoid pressure, and positive pressure ventilation before securing the patient's airway [3].

Rapid sequence induction is universally required in any occasion of emergent endotracheal intubation needed for unfasted patients or patients' fasting status is unknown like trauma patients, emergency surgery, resuscitation and patients with diminished level of consciousness, patients who are known for gastroesophageal reflux, diabetes, Parkinson's disease, gastric banding surgery, severe pain, recent opioid use, and pregnancy [4].

During rapid sequence induction, patients should be in optimal condition as much as possible, and they must not experience pain and major discomfort and recall to avoid any psychological trauma afterward [5].

Rapid sequence induction is the safest and fastest technique of induction to protect the airway from adverse airway events like pulmonary aspiration. During planning of this technique, there should be skilled assistance for the application of cricoid pressure [6].

Wrong technique of rapid sequence induction by most anesthetists results in high chance of failure to intubate, experiencing of regurgitation, hypoxia, and patient death [7].

Among many clinical skills applied to maintain the airway management both in the operating theater and out of the operating theater, the skill of applying safe and efficient rapid sequence induction is arguably one of the most frequently used and important techniques while encountering clinically indicated patients [8].

Cricoid pressure during the rapid sequence induction technique is the gold standard means of preventing gastric content entering the respiratory tract if it is applied correctly. But incorrect application of cricoid pressure leads to complications and risks to the patient [9].

To reduce adverse events to the patient during rapid sequence induction, it is important developing standards and procedural checklists [10]. In hemodynamically instable patients, using fentanyl, ketamine, and rocuronium produce the best intubating condition comparing the classic technique [11].

The incidence of desaturation during rapid sequence induction in Gondar University Hospital is around 35.9% which is too high clinically. The reason for the incidence is thought to be inadequate preoxygenation and lack of trained assistant [12].

This clinical audit is aiming at evaluating the current practice of RSII in the University of Gondar against the well-established standard and giving training for those who do have skill gap to use it.

## 2. Materials and Methods

### 2.1. Audit Design and Period

An institutional-based prospective observational study was conducted from December 2017 to January 2018.

### 2.2. Audit Area

This audit was conducted in the GUH obstetrics operation room and the main surgical operation room located in Gondar town, Northwest Ethiopia.

### 2.3. Audit Source Population

All patients planned for emergency and elective operation.

### 2.4. Audit Population

All elective and emergency adult or pediatric patients with a risk of pulmonary aspiration who were operated under general anesthesia with rapid sequence induction and intubation during the audit period.

### 2.5. Exclusion Criteria

Patients with a risk of pulmonary aspiration who are planned to undergo surgery with susceptible cervical spine fracture, patients with anticipated difficult intubation, and patients who are operated under regional anesthesia and sedation.

### 2.6. Audit Sampling Procedure

All consecutive elective and emergency adult or pediatric patients with a risk of pulmonary aspiration who were operated under general anesthesia with rapid sequence induction and intubation at the University of Gondar Hospital operation theaters who were eligible for the study based on the inclusion-exclusion criteria.

### 2.7. Data Collection Method

Data were collected by using a standard checklist. The checklist was primarily prepared in English language.

### 2.8. Data Quality Management

The collected data were checked for the completeness, accuracy, and clarity. Then necessary corrections were made accordingly to the standard checklist for the audit.

### 2.9. Data Processing and Analysis Procedures

After completion of data collection, the data were entered in Microsoft Excel for analysis.

Guidelines used as reference for this clinical audit were World Federation of Societies of Anesthesiologists, *Scandinavian Journal of Trauma, Resuscitation and Emergency Medicine*, *British Journal of Anesthesia*, National Institute of Health, and *World Journal of Emergency Medicine* (Table 1).

## 3. Results

### 3.1. Sociodemographic Characteristics of Respondents

A total of 35 patients were operated during the study period. Of these, 31 (88.57%) patients were adults and 4 (11.43%) patients were pediatrics. Most of the patients were emergency (29 (82.857%)), and the rest were elective (6 (17.142%)). Around 97.1% of the anesthetists prepared the necessary airway equipment as per the standard (Table 2).

All anesthetists preoxygenated their patients during planning of rapid sequence induction, and only 14.3% of the anesthetists used bougie or stylet to maximize the chance of success (Table 3).

**Figure 1:**
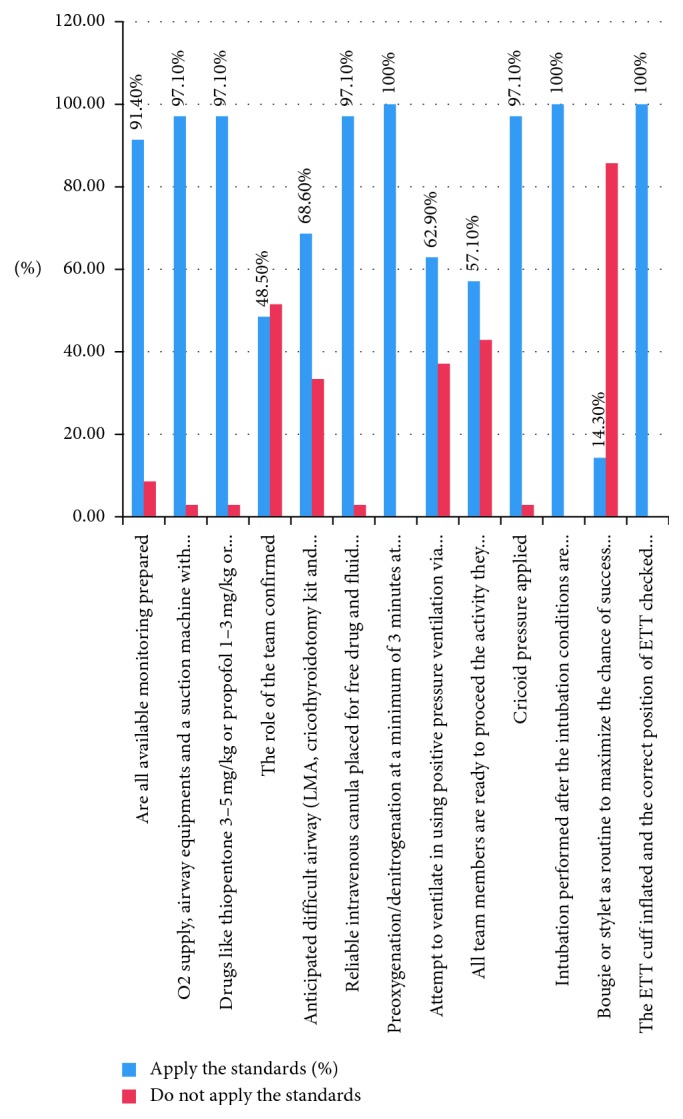
A bar graph shows current practice of rapid sequence induction and intubation in Gondar University Specialized Hospital, Northwest Ethiopia, 2018 (frequency) (*N* = 35).

Intubation performed after the intubation conditions are obtained after observing fasciculation and the ETT cuff inflated and the correct position of ETT checked by the chest rise and fall, tube misting, normal feeling of air flow or capnography, and releasing of cricoid pressure were applied by all anesthetists as per the standard (Figure 1).

## 4. Discussion

This clinical audit showed that the current practice of rapid sequence induction and intubation in GUH needs some improvement in some of the standards as recommended by the international guidelines.

Before intubating a patient with rapid sequence induction and intubation technique in this audit, around 48.5% of the anesthetists confirmed the role of the team what to do during the procedure, and around 57.1% of the anesthetists told to the team to do the activity they were assigned to. The intubator is the leader who also preoxygenates and administers drugs, while the assistant applies cricoid pressure and passes equipment to the intubator. A third person may be required for manual in-line stabilization of the neck if cervical spine injury is suspected [13].

Only around 14.3% of anesthetists in UOGH used bougie or stylet as a routine practice during rapid sequence induction to maximize the chance of success of intubation. Many scholars recommend the use of bougie or stylet as routine, and if available a video laryngoscope to maximize the chance of success of intubation is important [13]. In this clinical audit, 68.6% of anesthetists prepared difficult airway equipment [10].

Attempt to ventilate in using positive pressure ventilation via a face mask was applied by 62.9% of the anesthetists. Nowadays, some experts strongly recommend the routine use of positive pressure ventilation before tracheal intubation in certain RSII scenarios [3, 14].

If the standards are not applied correctly, it may result in desaturation, pulmonary aspiration, pneumonia, atelectasis, and finally death. The main reason for missing to apply the standards correctly may be secondary to lack of local working guidelines. Oxygen desaturation is the commonest complication for emergency patients, and greater emphasis should be put on formal preoxygenation as an essential part of RSI [1, 15].

This audit also showed that the practice of assigning the team for specific activity, preparation of difficult airway equipment like cricothyroidotomy kit, and attempt to ventilate patients during RSI is poor that needs some improvement [2].

## 5. Conclusion

### 5.1. Areas of Good Practice

Most anesthetists were good at preparing all available monitoring and drugs, making sure that IV line is well-functioning, preparing suction with a suction machine, preoxygenation, and application of cricoid pressure, and checking the position of the ETT after intubation is performed.

### 5.2. Areas Which Need to Be Improved

Preparing difficult airway equipment during planning of rapid sequence induction and intubation, giving roles and told to proceed the assigned role for each member of the team, attempt to ventilate with a small tidal volume, and routine use of bougie or stylet to increase the chance of success of intubation should be improved.

## Figures and Tables

**Table 1:** Standards of rapid sequence induction at risk of pulmonary aspiration at Gondar University Specialized Hospital, Northwest Ethiopia, 2018.

SN	Parameters/standards	Yes	No	Na
1	Are all available monitoring prepared			
2	O_2_ supply, airway equipment, and a suction machine with suction catheter prepared and placed on the table beneath the patient's head			
3	Drugs like thiopentone (3–5 mg/kg) or propofol (1–3 mg/kg) or ketamine 1-2 mg/kg for hemodynamically unstable patients, suxamethonium (1-2 mg/kg) and fentanyl (1-2 *μ*g/kg prepared)			
4	The role of the team confirmed			
5	Anticipated difficult airway (LMA, cricothyroidotomy kit, and oxygenation plan) prepared			
6	Reliable intravenous cannula placed for free drug and fluid administration			
7	Preoxygenation/denitrogenation at a minimum of 3 minutes at an oxygen concentration of 100% done.			
8	Attempt to ventilate in using positive pressure ventilation via a face mask			
9	All team members are ready to proceed the activity they were assigned to			
10	Cricoid pressure applied			
11	Intubation performed after the intubation conditions are obtained after observing fasciculation			
12	Bougie or stylet as routine to maximize the chance of success was used			
13	The ETT cuff inflated and the correct position of ETT checked by the chest rise and fall, tube misting, normal feeling of air flow or capnography, and releasing of cricoid pressure			

Na = not available.

**Table 2:** Preparation of equipment, monitoring, and anesthetic drugs at Gondar University Specialized Hospital, Northwest Ethiopia, 2018.

Standards	No. of anesthetists	Who meet standards	Who did not meet the standards	Applied standards (%)
Are all available monitoring prepared	35	32	2	91.4
O_2_ supply, airway equipment, and a suction machine with suction catheter prepared and placed on the table beneath the patient's head	35	34	1	97.1
Drugs like thiopentone (3–5 mg/kg) or propofol (1–3 mg/kg) or ketamine (1-2 mg/kg) for hemodynamically unstable patients, suxamethonium (1-2 mg/kg) and fentanyl (1-2 *μ*g/kg) prepared	35	34	1	97.1
The role of the team confirmed	35	17	18	48.5
Anticipated difficult airway (LMA, cricothyroidotomy kit, and oxygenation plan) prepared	35	24	11	68.6
Reliable intravenous cannula placed for free drug and fluid administration	35	34	1	97.1

**Table 3:** Pretreatment, induction medication, intubation, and confirmation at Gondar University Specialized Hospital, Northwest Ethiopia, 2018.

Standards	No. of anesthetists	Who meet standards	Who did not meet the standards	Applied standards (%)
Preoxygenation/denitrogenation at a minimum of 3 minutes at an oxygen concentration of 100% done	35	35	0	100
Attempt to ventilate in using positive pressure ventilation via a face mask	35	22	13	62.9
All team members are ready to proceed the activity they were assigned to	35	20	15	57.1
Cricoid pressure applied	35	34	1	97.1
Intubation performed after the intubation conditions are obtained after observing fasciculation	35	35	0	100
Bougie or stylet as routine to maximize the chance of success was used	35	5	28	14.3
The ETT cuff inflated and the correct position of ETT checked by the chest rise and fall, tube misting, normal feeling of air flow or capnography, and releasing of cricoid pressure	35	35	0	100

## Data Availability

Data and materials used in this study are available and can be presented by the corresponding author upon reasonable request.

## Abbreviations

ETT: Endotracheal tube
IV: Intravenous
LMA: Laryngeal mask airway
PPV: Positive pressure ventilation
RSI: Rapid sequence induction
UOGH: University of Gondar Hospital.

## Additional Points

*Recommendations*. We recommend that every anesthetist should give role to the team, attempt to ventilate patients with a small tidal volume in selected patient groups (critically ill, pregnancy, obesity, and pediatrics), and routinely use bougie or stylet to increase the chance of success of intubation.
